# A Unique Clinical Presentation of Congenital Thumb Aplasia, Radioulnar Synostosis, and Chiari Malformation: A Potential Pediatric Syndromic Association

**DOI:** 10.7759/cureus.66274

**Published:** 2024-08-06

**Authors:** Taylor F Faust, Jackson Carlyle, Julee Reitzel, Aftab Khan, Grace Williams

**Affiliations:** 1 Department of Research, Alabama College of Osteopathic Medicine, Dothan, USA; 2 Department of Research, Edward Via College of Osteopathic Medicine, Auburn, USA; 3 Department of Pediatrics, Decatur Morgan Hospital, Decatur, USA

**Keywords:** pediatrics orthopedics, vacterl syndrome, pediatric scoliosis, radioulnar synostosis, congenital thumb aplasia, chiari i malformation, pediatric genetics

## Abstract

The medical literature does not currently report a case of co-occurring congenital thumb aplasia, radioulnar synostosis (RUS), and Chiari malformation with scoliosis. Furthermore, there is an overlap of clinical features with other documented syndromes and associations that have potential cardiac, gastrointestinal, hematologic, and nephrological implications, thus contributing to increased morbidity and mortality if left undetected. We describe an interesting case of congenital thumb aplasia, RUS, and Chiari malformation with scoliosis in the absence of non-musculoskeletal abnormalities. These findings prompted further investigation to determine whether this is a unique presentation of a previously described syndrome, due to teratogenic exposure in utero, or a syndromic association yet to be adequately identified by the scientific community. We also identified several candidate genes that may guide genetic testing in the future.

## Introduction

When a set of clinical findings is remarkably diverse, it is imperative to determine whether the findings share a common origin. Congenital scoliosis has an estimated incidence of 0.5-1.0 per 1,000 people [1]. Congenital thumb aplasia has an estimated incidence of 1.0 per 100,000 people [2]. Fewer than 700 cases of congenital radioulnar synostosis (CRUS) have been documented [3]. Therefore, it is appropriate to suggest that it would be unlikely for these three findings to be co-occurring yet entirely unrelated. We need to investigate further to determine the potential causes of this symptomatology.

The number of processes that could have resulted in a syndromic association of scoliosis, radioulnar synostosis (RUS), and thumb aplasia is limited. The most likely cause would be genetic, either a sporadic or inherited mutation. We also considered other potential causes, such as teratogenic exposures, intrauterine infections, and gross developmental malformations due to the fetus’s positioning in utero.

Investigations into this clinical phenotype began with a review of the current literature surrounding appendicular malformations co-occurring with vertebral malformations and the syndromes characterized by thumb aplasia and RUS. Syndromes fitting this description include Fanconi anemia, Holt-Oram syndrome, and the VACTERL association (Vertebral anomalies, Anorectal malformations, Cardiovascular anomalies, Tracheoesophageal fistula, Esophageal atresia, Renal anomalies, Limb defects) [2].

It is crucial to consider the potential impact of maternal smoking on the development of this condition. Maternal smoking during pregnancy has been linked to a range of birth defects and developmental issues, including limb malformations [4]. Through Orphanet [5], we identified four candidate genes previously associated with all three primary abnormalities observed in our patient. The genes include CANT1, SALL4, SETBP1, and SMC1A. Additionally, a focused literature review highlighted SMAD6 as another potential candidate gene due to its strong association with RUS. Furthermore, we examined the BMP signaling pathway, its relation to SMAD6, and how this relates to our patient's presentation. 

## Case presentation

A male child, aged 29 months, presented to the outpatient pediatric clinic with left thumb aplasia, left-sided RUS, and scoliosis. Additional medical diagnoses included congenital torticollis, mild persistent asthma, and Chiari malformation. The patient was born at 37 weeks to a 20-year-old G4P4 mother, who had received prenatal care and was without maternal complications. The mother smoked one to three cigarettes a day during pregnancy. The child was delivered via C-section at 37 weeks due to breech presentation, weighing six pounds and two ounces, with a complication of hypertrophic pyloric stenosis, which was repaired following delivery. The patient’s family history was negative for intellectual disabilities, congenital disabilities, multiple miscarriages, early childhood deaths, or stillbirths. The father had a history of Crohn’s disease. The maternal grandmother had multiple sclerosis, and the paternal great-grandmother had colon cancer at 30 years old.

On the physical exam, the child appeared to be healthy. He was alert and not in any acute distress. The patient’s musculoskeletal examination showed a four-ray left hand with an absent left thumb. The patient was without pronation and supination of the left forearm. Examination of the spine revealed a dextroconvex deformity of the thoracic spine.

Radiographic exam

**Figure 1:**
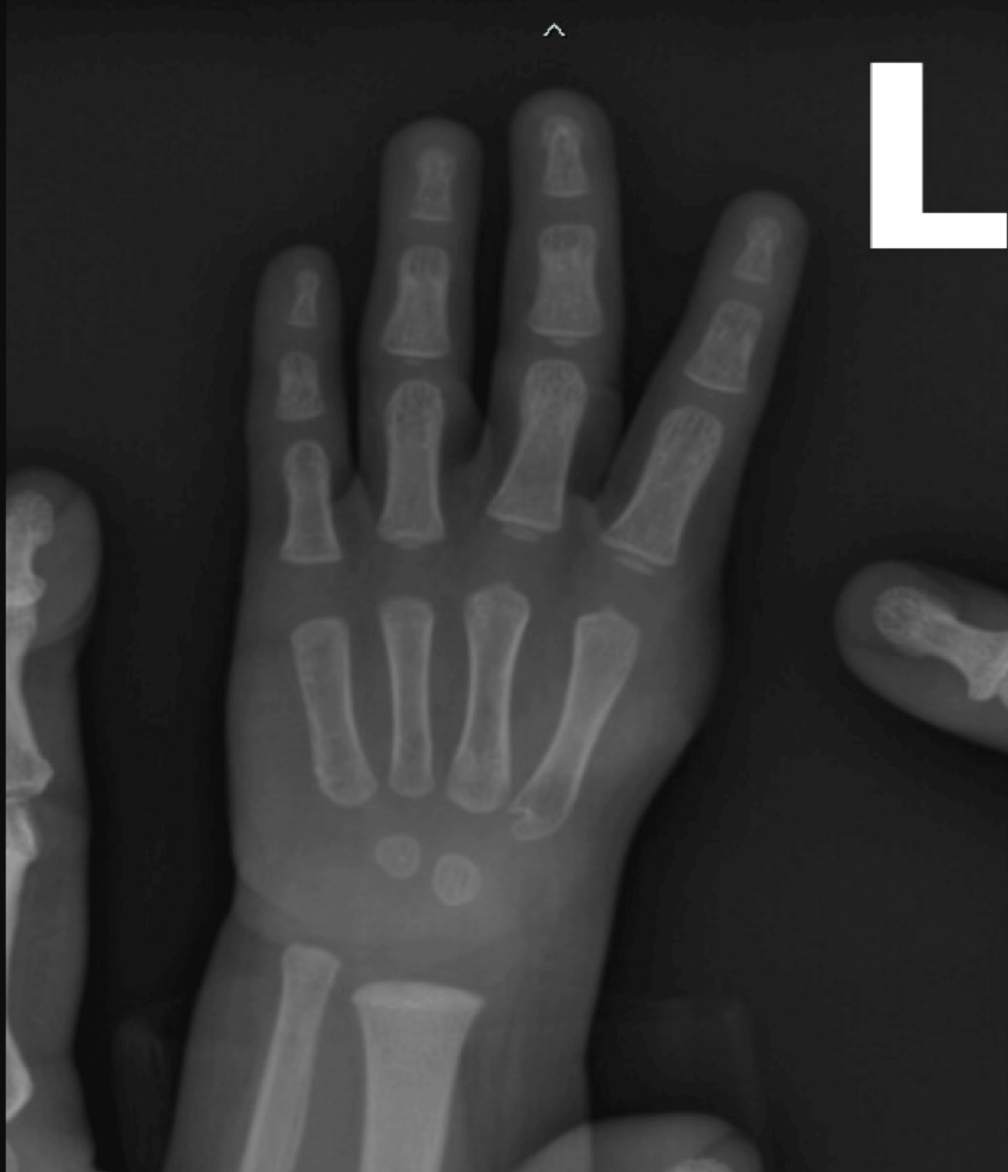
Left-hand X-ray The left thumb is absent, but the remainder of the hand is intact, and soft tissues are overall clear.

In the left-hand X-ray (Figure 1), the left thumb is absent; the remainder of the hand is intact; and the soft tissues are overall clear.

**Figure 2:**
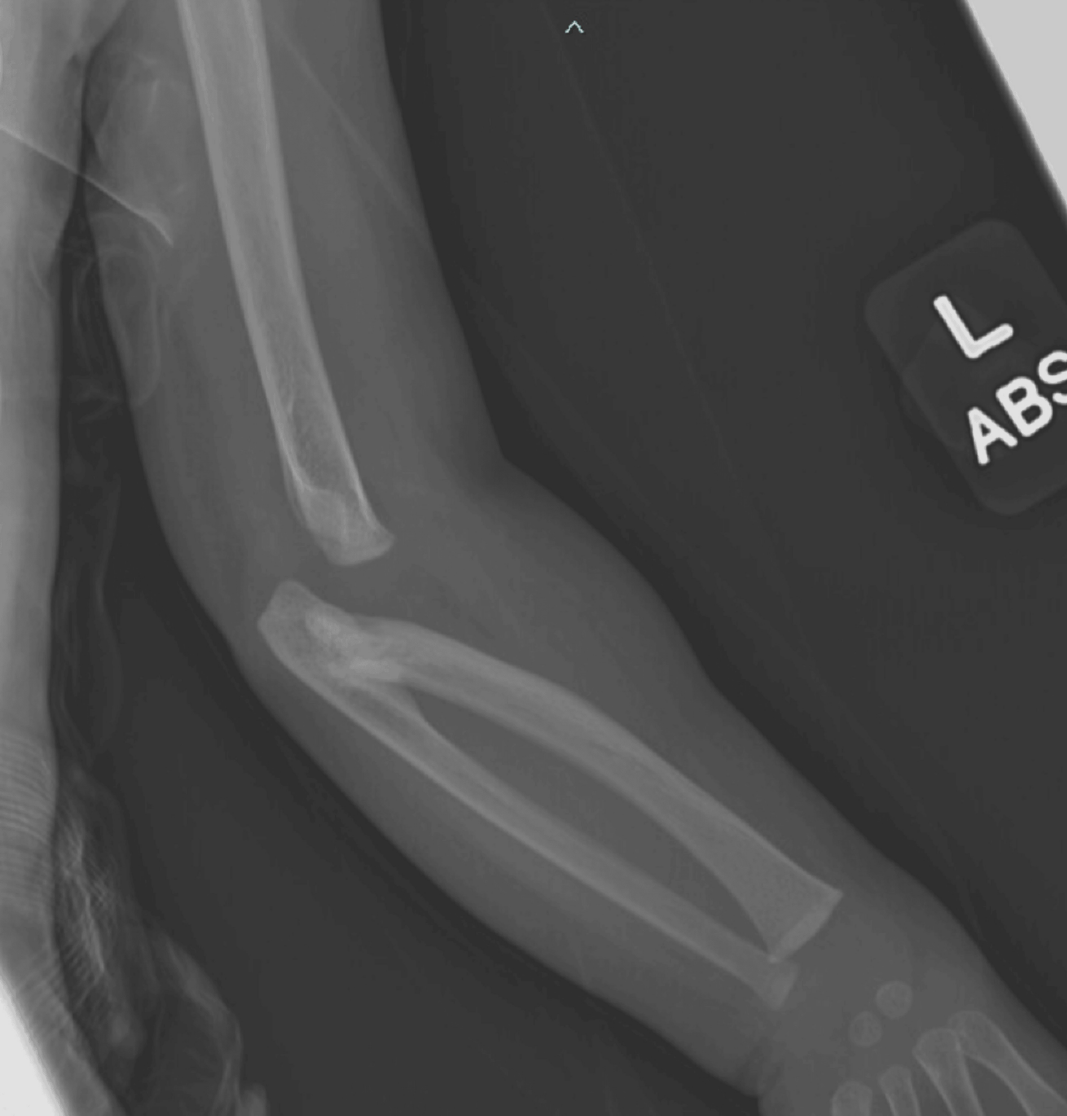
Left-forearm X-ray The distal humerus is dislocated from the radius and ulna. The radial head and olecranon appear to be malformed.

In the left forearm X-ray (Figure 2), the distal humerus is dislocated from the radius and ulna. Left RUS is identified by the fusion of the proximal radius to the proximal ulna and by the malformed radial head and olecranon.

**Figure 3:**
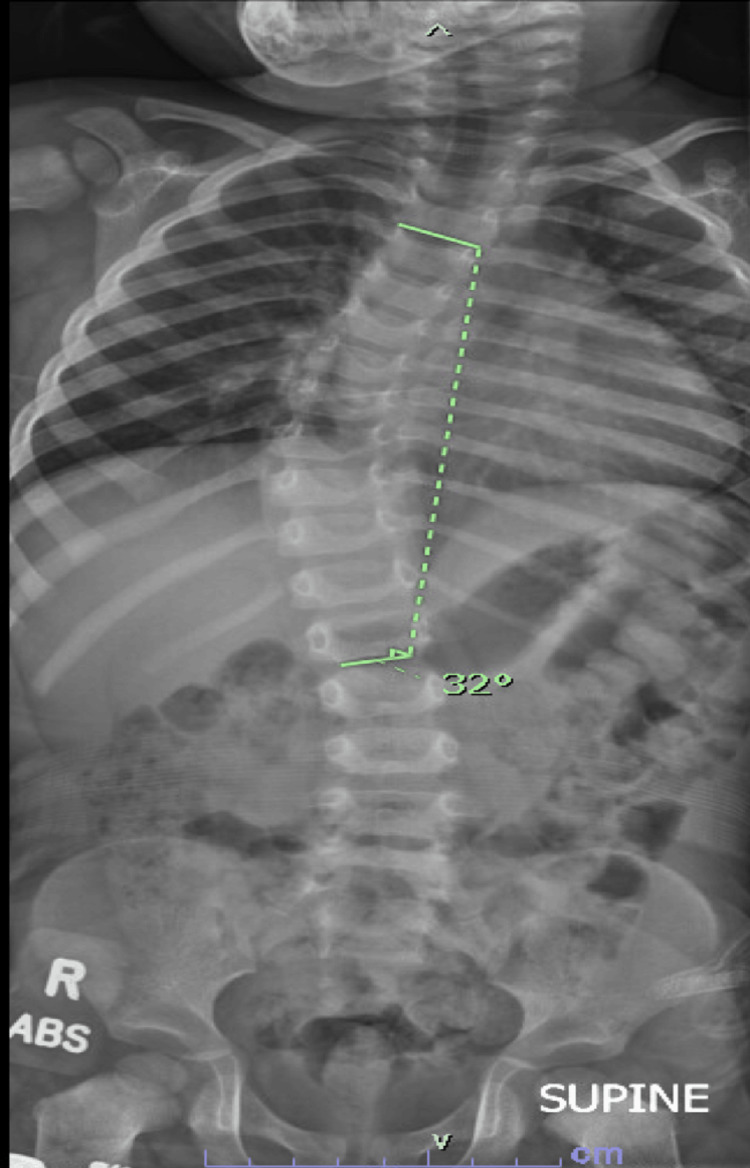
Spine anterior-posterior X-ray for scoliosis There is moderate dextroscoliosis of the thoracolumbar spine. This measures about 32 degrees and features moderate rotatory scoliosis tearing.

The anterior-posterior X-ray of the spine for scoliosis (Figure 3) was evaluated, revealing moderate dextroscoliosis of the thoracolumbar region (T4 to T12). The individual is undergoing bracing for realignment.

An MRI of the cervical, thoracic, and lumbar spine, with and without contrast, was completed. The mid-to-lower cervical spine showed minimal saccular hydromyelia or syrinx. A renal ultrasound of the left and right kidneys was unremarkable for abnormalities in both kidneys. Cardiovascular testing was completed with a transthoracic echocardiogram, 2D with spectral Doppler, and color flow Doppler. Results showed a structurally normal heart and unremarkable echocardiogram findings. A Fanconi anemia workup was conducted at an outside institution via stress testing and was negative. The Invitae Limb and Digital Malformations Panel was also negative. This panel tests for a wide range of genetic conditions that can cause limb and digital malformations. A negative result suggests that the patient’s condition is not likely caused by a known genetic syndrome associated with limb and digital malformations. Otherwise, the patient met all motor and mental milestones and was on track for a growth trajectory.

## Discussion

Approximately 6,000 to 9,000 known rare diseases affect 1 in 10 adults and children in Europe, with similar numbers in the U.S. [6]. On average, patients spend five years searching for a diagnosis, resulting in significant care delays and financial burdens [6]. Based on the findings and testing completed so far, the skeletal abnormalities observed in this patient collectively describe an undocumented presentation. In the absence of concurrent congenital anomalies and malformations, and with negative genetic testing results, it is imperative to thoroughly delineate the patient’s presentation and examine distinctions from other known syndromes. This report aims to raise awareness of a potentially new syndrome or shed light on an uncommon presentation of a known rare disease.

Musculoskeletal anomalies

Congenital absent digits are a phenotypic classification system designed for the morphological or radiographic characteristics observed in individuals with hand anomalies, known as Blauth’s classification system [7]. It was explicitly designed to identify and categorize children with such anomalies. Multiple categories vary in severity. Type I is “minimal shortening and narrowing,” whereas Type V is “complete thumb absence” [8]. The patient presented was classified as Type V. This system can be utilized to classify congenital missing digits. 

The patient’s other musculoskeletal abnormality in this case report is RUS. CRUS is a rare malformation involving an abnormal connection between the radius and ulna due to embryonic separation failure [7]. The Cleary-Omer classification outlines four types of proximal RUS (PRUS). Type I involves a fibrous connection between the proximal ulna and radius, whereas Type II features an osseous connection, with the radial head in a normal position. Type III involves the fusion of bones (osseous synostosis) and backward displacement of the underdeveloped radial head (posterior dislocation). Type IV is characterized by an anterior dislocation of a mushroom-shaped radial head, accompanied by a fibrous pseudo-synostosis [9]. Our patient would most likely be classified as Type III based on radiographic findings. 

Bai et al. looked at treatment for CRUS and mentioned one case of ipsilateral thumb hypoplasia and one case of bilateral thumb hypoplasia [7]. Additionally, other studies on CRUS have reported its association with various syndromes, including amegakaryocytic thrombocytopenia, Carpenter syndrome, and Holt-Oram syndrome [10,11]. Although the patient outlined in this case report had limb abnormalities alongside RUS, the reviewed study highlights the occurrence of this deformity in conjunction with thumb hypoplasia, similar to our patient. This observation suggests a connection or interrelationship between the two phenomena.

Because of their likely interrelation, we will consider Chiari malformation and scoliosis as interconnected structural abnormalities. UpToDate shows that scoliosis is one of the most common presentations of Chiari malformation in this age group [12]. A few syndromes include Chiari malformation and thumb abnormalities, and there is only one recorded instance of Chiari malformation occurring with RUS [13]. To date, there is no record of these abnormalities co-occurring.

Syndrome and association anomalies

VACTERL association is a condition characterized by vertebral abnormalities and appendicular abnormalities; however, it is also commonly characterized by a group of medical abnormalities not observed in this patient but worth mentioning [14]. These primarily include anal atresia, cardiac defects, tracheoesophageal abnormalities, and renal abnormalities [14]. It should be noted that our patient underwent surgery to correct pyloric stenosis. Previous cases of VACTERL association have described this anomaly, even though hypertrophic pyloric stenosis is not a typical feature of VACTERL [14]. Furthermore, VACTERL is a diagnosis of exclusion, diagnosed when a patient has three of the associated findings and all other genetic testing is negative [14]. Further complicating our patient’s diagnosis is the fact that the mother smoked cigarettes during pregnancy, which is associated with hypertrophic pyloric stenosis [4]. Because of this complication, it is unclear whether our patient meets the criteria for VACTERL association. RUS and thumb aplasia are manifestations of abnormal limb development that can be a part of VACTERL. However, depending on the definition of VACTERL used, radial defects could stand alone or be included among the limb defects. 

Another syndrome worth mentioning is Holt-Oram syndrome, which typically results in deformities of the upper limbs, congenital cardiac defects, and conduction abnormalities [15]. Although this patient had not been diagnosed with cardiac abnormalities, these defects can manifest later. Holt-Oram syndrome occurs due to a mutation in the TBX5 gene. However, our patient had a negative Invitae Limb and Digital Malformations Panel, which most likely included testing for the TBX5 mutation [15].

Fanconi anemia is another syndrome of note. However, the absence of clinical features rules out this syndrome in the patient. Fanconi anemia usually presents in a child’s first decade of life upon recognition of aplastic anemia. It features thumb and radial absence, malformation, or even a deeper cleft between the first two digits. Other notable features include skin discolorations, abnormal reproductive organs, a small head or eyes, kidney problems, low birth weight, heart effects, and gastrointestinal issues [16]. The patient underwent stress testing, which was negative. If the patient exhibits lab abnormalities in the future or if there is concern for Fanconi anemia, the medical team should consider culturing the patient’s skin fibroblasts to rule out mosaicism. 

Thumb aplasia syndromes

Two other notable syndromes highlighted by thumb hypoplasia are Yunis-Varón syndrome and Nager syndrome. Yunis-Varón syndrome is a rare autosomal recessive condition that features the absence of thumbs and the presence of hallucinations, distal phalanges, and ectodermal anomalies, along with growth retardation and poor outcomes. Defective growth of the cranial bone characterizes the condition, resulting in craniofacial disproportion, which can include complete or partial absence of the clavicles (cleidocranial dysplasia) [17]. Nager syndrome is a rare autosomal recessive syndrome characterized by craniofacial malformations, including downward-slanting palpebral fissures, bilateral lower eyelid colobomas with absence of cilia of the lower eyelids, malar hypoplasia, a broad and high nasal bridge, micrognathia, retrognathia, malformations of the auricular pinna, and atresia of the auditory meatus of the right ear. This condition also includes bilateral conductive hearing loss and a cleft palate with an absent soft palate. Nager syndrome’s limb abnormalities typically include hypoplastic or absent thumbs and radii [18]. These studies illustrate thumb aplasia, a notable feature in our patient’s case. However, our report delineates a unique presentation because the patient lacks the other characteristics of Yunis-Varón syndrome and Nager syndrome. Further, the patient’s genetic tests were negative, making unusual presentations of these disorders even more unlikely.

Maternal cigarette smoking

This patient’s mother had limited tobacco exposure during pregnancy (one to three cigarettes per day), but it must be emphasized that cigarette smoking is associated with several congenital malformations, including limb defects [4]. Although there is no direct link between Chiari malformations and maternal cigarette smoking in the literature, maternal tobacco exposure has been linked to neural tube and skull defects [4]. It is also worth noting that hypertrophic pyloric stenosis is associated with cigarette smoking [4].

Genetic outcome

Because of the absence of a formal diagnosis for our patient, based on clinical history and negative genetic testing, we felt that it was advisable to explore specific genes that may be associated with this rare clinical manifestation. We utilized Orphanet [5], a comprehensive database for rare diseases, to identify genes associated with the three primary abnormalities observed in this patient, namely thumb aplasia, RUS, and scoliosis. The results from each query were cross-referenced, leading to the identification of four candidate genes selected for further investigation: CANT1, SALL4, SETBP1, and SMC1A.

Moreover, a literature review identified the SMAD6 gene as a significant factor contributing to RUS [19]. SMAD6 is mutated in 42.11% of RUS pedigrees and 15.52% of RUS sporadic patients [19]. Additionally, when the SMAD6 variant is the cause of congenital RUS, the left side is more susceptible than the right side [19]. In our patient, the RUS occurred on the left, thumb aplasia was on the left, and the scoliosis was dextroscoliosis, meaning the spine was bent to the right.

Another gene-related system discovered in the literature review is the bone morphogenetic protein (BMP) signaling pathway, which is essential for the induction of bone formation, interdigital cell death, axial skeleton formation, embryogenesis, and the development of various organ systems [20]. SMAD6, as mentioned above, along with the gene NOG (encoding the noggin protein), is heavily involved in the BMP signaling pathway [20].

Regular echocardiographic evaluations are essential for patients who test positive for the SMAD6 variant. Severe cardiovascular issues may be associated with SMAD6, BMP, and the development of cardiac structures [20].

In line with the management of patients with other syndromic associations, close monitoring and regular echocardiograms would be the most appropriate approach for the patient in this report.

Limitations

One limitation of this report is that we were unable to access the detailed Invitae Limb and Digital Malformation report. Thus, it is unclear whether some genes, such as NOG, SMC1A, TBX5, and SALL4, were all included. Clinical documentation from outside institutions was reviewed, and this panel was reportedly negative.

## Conclusions

Based on the findings presented in this report, the patient does not align with the criteria for several existing syndromes associated with the features of thumb aplasia, radioulnar synostosis, and Chiari malformation with scoliosis. Conducting available and future genetic tests holds promise for a more targeted investigation. Utilizing additional rare disease databases and future medical knowledge may help identify the specific syndrome indicated in our patient. Some noteworthy genes discussed in this report, including SMAD6, NOG, and those associated with the BMP pathway, are starting points for further exploration. While these genes may be evaluated at a later time, the patient is currently being monitored for new clinical manifestations or abnormalities that could suggest a potential known syndrome.

Continued clinical follow-up is essential to manage and watch for new findings that may contribute to meeting the criteria for an existing syndrome. This anomaly represents a singular case, but understanding its genetic underpinnings may help improve prognosis and inform preventive and early intervention strategies for future patients with similar features. Given the other skeletal anomalies discussed, ongoing cardiovascular investigation with routine echocardiography is imperative and should be maintained as the patient transitions into adulthood. Continued monitoring and inquiry about the potential emergence of unforeseen anomalies or progression of related systems remain of significant interest as the individual matures.
